# Treatment of Upper Lumbar Disc Herniation with a Transforaminal Endoscopic Technique

**DOI:** 10.3389/fsurg.2022.893122

**Published:** 2022-04-28

**Authors:** Zuowei Wang, Fengzeng Jian, Hao Wu, Xingwen Wang, Kai Wang, Wanru Duan, Zhenlei Liu, Zan Chen

**Affiliations:** Department of Neurosurgery, Xuanwu Hospital, Capital Medical University, China

**Keywords:** high level, upper lumbar disc herniation, Minimally invasive, endoscope, percutaneous endoscopic transforaminal discectomy

## Abstract

**Background:**

To investigate the clinical efficacy of percutaneous endoscopic transforaminal discectomy (PETD) in the treatment of upper lumbar disc herniation (LDH).

**Methods:**

Twenty-two patients, 14 males and 8 females with ages ranging from 23 to 76 years, who had upper LDH and were treated with PETD from April 2015 to April 2020 in the Department of Neurosurgery of Xuanwu Hospital, were selected to evaluate the surgical efficacy by the visual analog scale (VAS) and Oswestry Disability Index (ODI).

**Results:**

All patients underwent successful completion of PETD surgery. The operation time was 80.4 ± 18.0 min; intraoperative fluoroscopy was used 17.1 ± 8.7 times; and the hospital stay was 3.2 ± 0.6 days. The VAS scores were 7.9 ± 1.2, 2.3 ± 1.5, 2.2 ± 1.3, and 2.1 ± 1.0 before the operation, 1 day and 3 months after the operation, and during the last follow-up, respectively. The postoperative VAS score was significantly lower than that before the operation (*P* < 0.01). The ODI scores before and 3 months after the operation were 59.8 ± 16.8 and 15.3 ± 8.2, respectively; thus, the postoperative score was decreased (*P* < 0.01).

**Conclusion:**

Upper lumbar discs have unique anatomical structures, and PETD is a safe and effective surgical method for the treatment of upper LDH.

## Introduction

Upper lumbar disc herniation (LDH) refers to an annulus fibrosus rupture or a herniated nucleus pulposus at or above L3–L4, and some scholars suggest that upper LDH refers to the L1–L2 and L2–L3 levels (1, 2). The incidence of upper LDH is low, accounting for approximately 5% of all LDH cases (3). Compared with lower LDH, upper LDH has unique anatomical characteristics, including a narrow spinal canal volume, narrow distance between the nerve roots and dura mater, shorter nerve roots in the intervertebral foramen area, and close proximity to the conus medullaris. Therefore, upper lumbar intervertebral disc surgery carries a higher risk, and the surgical results are not satisfactory (4).

With the improvement in spinal endoscopic technology, the efficacy of percutaneous endoscopic transforaminal discectomy (PETD) in the treatment of LDH has become comparable to that of open surgery. In addition, this technique involves less surgical trauma, faster postoperative recovery, and no adverse effects on spinal stability and has been widely used (5). At present, some clinicians use spinal endoscopy for upper discectomy. Due to the unique properties of upper LDH, its endoscopic treatment has different characteristics from those of lower LDH (6). In this study, we summarized and analyzed the clinical data of patients with upper LDH treated with PETD to explore the operating skills and clinical efficacy of the surgery.

## Materials and Methods

### General Data

Twenty-two patients with upper LDH (3 L1–L2 cases, 6 L2–L3 cases, and 13 L3–L4 cases) treated with PETD from April 2015 to April 2020 in the Department of Neurosurgery of Xuanwu Hospital, China, were selected, including 14 males and 8 females; the age ranged from 23 to 76 years, with an average of 44.3 years; the disease course ranged from 1 to 23 months, with an average of 5.2 months.

Clinical manifestations: radiating pain in the lower extremities in 21 cases, lumbosacral pain in 11 cases, numbness in the area where affected nerves were distributed in 9 cases, lower extremity weakness in 3 cases, perineal pain in 3 cases, perineal numbness in 2 cases, and hip pain in 2 cases.

Inclusion criteria: (1) First time surgery; (2) single-segment LDH at L1–L2, L2–L3, or L3–L4 or LDH combined with spinal stenosis; (3) radicular pain and low back pain associated with disc herniation; (4) poor results or frequent relapse after conservative treatment for more than 4 weeks.

Exclusion criteria: patients undergoing reoperation, and cases involving severe disc calcification, severe significant lumbar degenerative deformity, segmental instability, bony spinal stenosis, or cauda equina syndrome.

### Imaging Data

All patients underwent routine preoperative examinations by lumbar computed tomography (CT), magnetic resonance imaging (MRI), and lumbar anteroposterior, lateral, hyperextension, and hyperflexion X-ray. The imaging examinations were used to confirm the diagnosis and type of LDH, shape and size of the intervertebral foramen, height of the iliac crest, and shape of the spine and to determine lumbar spine stability.

Classification according to the site of protrusion: 5 cases of central protrusion and 17 cases of paramedian protrusion. Classification according to pathology: 18 cases of protrusion type, 3 cases of prolapse type, and 1 case of sequestered type.

### Surgical Methods and Perioperative Management

**Figure 1:**
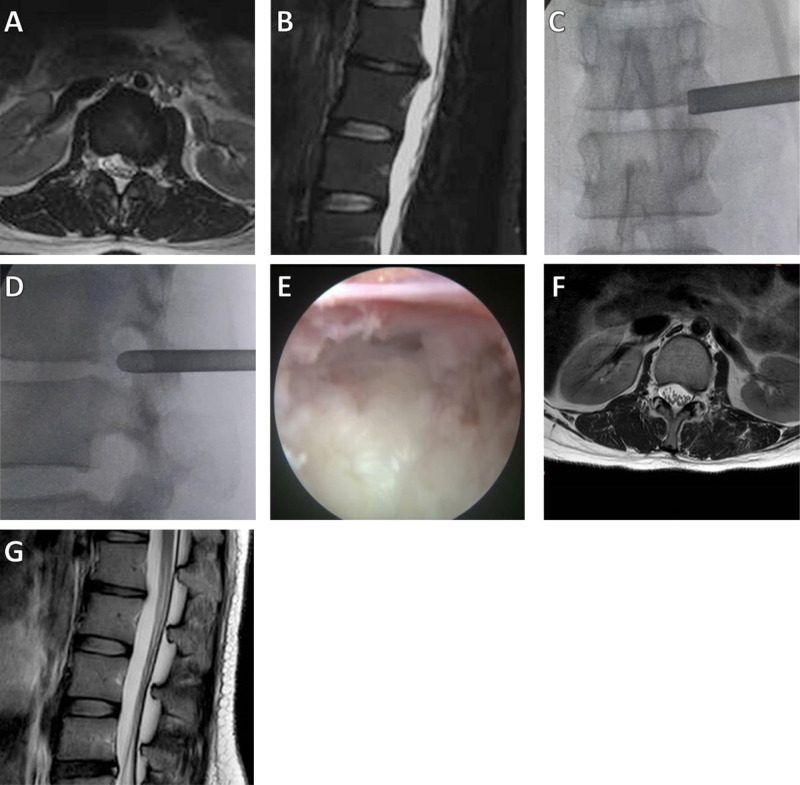
A 28-year-old female patient had radiating pain in the left lower extremity. (**A**,**B**) Preoperative axial and sagittal MRI results showed L1–2 LDH, with compression of the dural sac; (**C**,**D**) Intraoperative anteroposterior and lateral X-rays show the position of the working cannula; (**E**) The herniated intervertebral disc was removed, and the nerve root decompression was satisfactory; (**F,G**) Postoperative MRI showed that the herniated intervertebral disc resection was satisfactory.

(1) Surgical methods: Combined local anesthesia and intravenous anesthesia was used. The patient was placed in the lateral decubitus position. According to the patient’s body size and the operation segment, the puncture site was approximately 6–10 cm from the midline, and the puncture direction was caudally inclined by 5–30°. The subcutaneous tissue, deep fascia, and facet joints were anesthetized by local infiltration of lidocaine and ropivacaine. After the target disc was positioned under fluoroscopy, the positioning needle was inserted into the base of the superior articular process of the lower vertebral body. The soft tissue expansion cannula and working catheter were inserted sequentially along the guide wire (if necessary, intervertebral foramen formation was performed under the visualization channel, and part of the inner wall of the superior facet was removed with a trephine) (**Figure 1**).

After the successful placement of a working channel, a foraminoscope (SPINENDOS, Germany) was inserted. The herniated, prolapsed, or sequestered intervertebral disc tissue was removed using grasping forceps under direct vision through the foraminoscope, and part of the hypertrophic or calcified ligamentum flavum was removed or trimmed. Finally, the ruptured annulus fibrosus was ablated and shrunken using bipolar radiofrequency. When the nerve root was fully decompressed, the endoscope and working cannula were removed. The subcutaneous tissue and the wound were sutured.

(2) Perioperative management: Broad spectrum antibiotics were used once during surgery. Patients were allowed to get out of bed after 4 to 18 h of bed rest following the surgery. Patients wore a soft waist brace for 3 weeks after the surgery and avoided excessive physical activity and strenuous physical exercise for 3 months.

### Efficacy Evaluation

Evaluation with the visual analog scale (VAS) was performed before the operation, 1 day and 3 months after the operation, and during the last follow-up (7). Evaluation with the Oswestry Disability Index (ODI) was performed before the operation and 3 months after the operation to assess the improvement in pain after the operation. The improvement rate = (preoperative ODI score−last follow-up ODI score)/preoperative ODI score × 100%. An improvement rate of 75%–100% was considered excellent, 50%–75% was considered good, 25%–49% was considered fair, and <24% was considered poor. An improvement rate of >25% was considered effective. Lumbar MRI was reexamined one day after the operation, and lumbar MRI, CT, and X-ray results were reexamined 3 months and 1 year after the operation to observe the presence or absence of residual nucleus pulposus or LDH recurrence and the stability of the spine.

### Statistical Methods

The data were analyzed using SPSS version 23.0 (SPSS Inc., Chicago, IL, USA), and are represented by $\bar{x}\pm s$. The preoperative and postoperative VAS and ODI scores were compared using a paired t-test, and *P* < 0.05 was considered statistically significant.

## Results

All 22 patients completed surgical treatment with PETD, and the results were as follows.

1. Surgical results: The operation time was 50–125 min, with an average of 80.4 ± 18.0 min; the intraoperative blood loss was minimal; therefore, it was not evaluated; intraoperative fluoroscopy was used 11–45 times, with an average of 17.1 ± 8.7 times; the postoperative hospital stay was 2–5 days, with an average of 3.2 ± 0.6 days.
2. Complications: One patient had decreased thigh flexion muscle strength on the affected side after the operation, resulting in a decrease from the preoperative grade of 5 to 2. The patient underwent rehabilitation exercise therapy, and the strength recovered to grade 4 after 1 month and to grade 5 after 3 months. One patient had postoperative numbness of the lower extremity on the diseased side and recovered after 3 weeks. No surgical complications such as intervertebral space infection occurred.
3. Follow-up results (**Table 1**): All 22 patients had preoperative and postoperative VAS and ODI scores. Twenty-one patients (95.5%) were effectively followed up for more than 12 months, and the follow-up time ranged from 12 to 47 months, with an average of 19.7 months. The VAS scores were 7.9 ± 1.2, 2.3 ± 1.5, 2.2 ± 1.3, and 2.1 ± 1.0 before the operation, 1 day and 3 months after the operation, and during the last follow-up, respectively. The postoperative VAS score was significantly reduced compared to the preoperative value (all *P* < 0.01). The ODI scores before the operation and 3 months after the operation were 59.8 ± 16.8 and 15.3 ± 8.2, respectively; therefore, the postoperative value was lower than the preoperative value (*P* < 0.01). Evaluation of the efficacy according to the improvement rate: excellent in 18 cases (81.8%), good in 2 cases (9.0%), fair in 1 case (4.5%), and poor in 1 case (4.5%), with an excellent and good rate of 90.9% and an effective rate of 95.5%. There were no cases of recurrence.

**Table 1:** Difference between pre- and postoperative scores $\left(\bar{x}\pm s\right)$ for patients with lumbar disc herniation.

Date	VAS score (points)	ODI score (points)
Before surgery	7.9 ± 1.2	59.8 ± 16.8
1 day after surgery	2.3 ± 1.5^a^	
3 months after surgery	2.2 ± 1.3^a^	15.3 ± 8.2^a^
Final follow-up	2.1 ± 1.0^a^	

*Note: ^a^Compared with preoperative VAS and ODI, P = 0.000.*

*VAS, visual analog scale; ODI, Oswestry disability index.*

## Discussion

Compared with the lower lumbar spine (L4–L5 and L5–S1), the incidence of upper LDH is lower, accounting for approximately 5% of LDH, with herniation occurring at L3 to L4 accounting for approximately 70%–83% of cases (1). The lower incidence of upper LDH may be due to the upper lumbar spine having less movement, and the relative stability of the lumbar spine reduces lumbar disc degeneration, thus reducing LDH (8, 9).

The anatomical structure of the upper lumbar vertebral body and accessories is quite different from that of the lower lumbar spine. The vertebral bodies and intervertebral discs of the upper lumbar vertebra are relatively small, and the spinal canal mainly has an oval shape, with no or a very shallow lateral recess. The epidural space is small, and the fat content of the epidural space is very low. The surrounding anatomical environment lacks buffer space. Additionally, there are more nerve tissues in the dura, and the nerve roots are short and tend to run horizontally (5, 10). Therefore, once disc herniation occurs, even if the degree is very mild, it can still cause significant compression of the spinal cord and result in corresponding symptoms. Disc herniation does not directly compress a single nerve root but compresses the dural tissue, causing complex and diverse clinical manifestations. Few patients exhibit upper LDH; therefore, misdiagnosis and missed diagnosis can easily occur. The patients in this study had radiating pain in the lower extremities, lumbosacral pain, limb numbness, lower limb weakness, perineal pain, perineal numbness, hip pain, and other symptoms. The locations of their pain were extensive, and most of the patients had more severe low back pain symptoms. There were few typical signs of nerve root localization similar to lower LDH, while many patients showed symptoms of cauda equina compression. Among the patients in this study, 5 had bilateral symptoms without the intermittent claudication symptoms of spinal stenosis. Upper LDH is easily confused with other diseases, and it requires the attention of clinicians.

When upper LDH occurs, there is little buffer space after the nerve and spinal cord are compressed, and the symptoms often cannot be relieved by themselves. For patients that fail to respond to conservative treatment, surgical treatment should be carried out. Traditional surgical methods include lumbar microdiscectomy and lumbar discectomy combined with intervertebral fusion (11). Open surgery for the treatment of upper LDH has a satisfactory clinical effect, but the operation requires extensive traction and dissection of paravertebral soft tissues, which tends to impair the stability of intervertebral joints and ligaments and results in increased surgical trauma (5). With improvements in spinal endoscopic techniques, the PETD technique has been widely used in the treatment of LDH, and the surgical efficacy has become comparable to that of open surgery. In addition, the surgical trauma is reduced and the postoperative recovery time is decreased (12). At present, PETD has been studied for the treatment of upper LDH (6, 13, 14). The upper lumbar lamina space is relatively narrow and the foramina is relatively large, so PETD is superior to percutaneous endoscopic interlaminar discectomy (PEID). In this study, PETD was used to treat upper LDH, and good results were obtained. The excellent and good rate was 90.9%, and the effective rate was 95.5%. Except for one patient with decreased muscle strength after the surgery, the operation was successfully completed in the remaining patients. Compared with traditional open surgery, PETD has a shorter operation time, less blood loss, fewer wound complications, and less postoperative instability. This is because endoscopic surgery reduces paravertebral muscle injury and preserves the posterior ligament and bone structure, thereby reducing iatrogenic tissue trauma. The operation was completed in all patients under local anesthesia, and patients were allowed to get out of bed 4 h after the operation. The degree of postoperative pain relief and wound healing in patients were faster than those in patients undergoing open surgery, and the patients could return to normal work after 3 weeks of rest.

Because the upper lumbar spine has different anatomical characteristics from those of the lower lumbar spine, the key points in performing endoscopic surgery are also different from those of the lower lumbar spine. Compared with the lower lumbar, the foramina of the upper lumbar vertebrae is larger. Thus the placement of the working channel is relatively simple and foraminoplasty is not required. As a result, the operation time of the upper lumbar TELD is shorter and the operation is easier. The upper lumbar spinal canal is small, there is less epidural fat, and the nerve roots emanate from the dural sac at the level of the middle and lower 1/3 of the vertebral body, leaving the intervertebral foramen below the corresponding pedicle. In addition, there are more nerve tissues in the dura mater at this site, the buffer space in the spinal canal is small, and the risk of damaging the dural sac and nerve roots during surgery is increased (6, 15). To prevent nerve damage, in this study, when performing a puncture to establish a working channel, the puncture point was located closer to the posterior midline. Skin puncture was performed 6–8 cm from the posterior midline, and the angle of the puncture reached approximately 40°. The puncture point for the L1–L2 segment was closer to the midline than those for the L2–L3 and L3–L4 segments. With this puncture method, nerve roots and the dural sac can be avoided, and the internal organs are not easily injured. There are many neurovascular variations in the upper lumbar spine, and it is necessary to adjust to the patient’s response during surgery. If the patient has radiating pain or weakness in the lower extremities, the puncture direction must be changed in a timely manner to prevent permanent nerve injury. When an even larger puncture angle is used, the established working channel is usually positioned more to the outside, and the surgeon does not operate directly in the spinal canal. First, part of the intervertebral disc is removed, and with progression of the surgery, a larger space is obtained; then, the operation is performed in the spinal canal. When upper lumbar discectomy is performed, it is more likely to damage the blood vessels accompanying the nerve roots, thus causing increased blood loss. Therefore, it is necessary to operate as close to the lower edge and the ventral side of the intervertebral foramen as possible. When larger blood vessels obscure the surgical field, they should be cauterized in advance.

Compared with the lower lumbar spine, upper lumbar is more stable and the discectomy recurrence rate is low. In addition to resecting the nucleus pulposus protruding into the spinal canal, further removal of the nucleus pulposus tissue in the intervertebral disc is not needed. However, the loose nucleus pulposus in the intervertebral disc should be removed. Allowing the patients to cough or hold their breath can help further distinguish the potential protruding nucleus pulposus. There were no cases of recurrence in this study.

Due to the low incidence rate, only 22 patients were included in this study, which is a limitation of the study. More patients should be included in future studies, and long-term follow-up should be performed.

## Conclusion

Because of the unique anatomical structure of the upper lumbar spine, patients with upper LDH have unique clinical manifestations, and surgical treatment also has unique characteristics. PETD is an effective method for the treatment of upper LDH.

## Data Availability

The original contributions presented in the study are included in the article/supplementary material, further inquiries can be directed to the corresponding author/s.
